# Critical Dialogue and Capacity-Building Projects Reduced Alcohol and Substance Use in a Randomized Clinical Trial Among Formerly Incarcerated Men

**DOI:** 10.1080/10826084.2024.2352611

**Published:** 2024-06-19

**Authors:** Liliane Cambraia Windsor, Ellen Benoit, Carol Lee, Alexis Jemal, Kari Kugler, Douglas C. Smith, Rogério M. Pinto, Salma Musaad

**Affiliations:** a School of Social Work, The University of Illinois, Urbana-Champaign, Illinois, USA; b North Jersey Community Research Initiative, Newark, New Jersey, USA; c Department of Psychiatry, Michigan Medicine, The University of Michigan, Ann Arbor, Michigan, USA; d City University of New York, Silberman School of Social Work, Hunter College, New York, New York, USA; e College of Health and Human Development, University of Pennsylvania, College Station, Pennsylvania, USA; f Baylor College of Medicine, Waco, Texas, USA

**Keywords:** Substance use intervention, critical consciousness, multiphase optimization strategy, community-based participatory research, urban predominantly black and marginalized communities

## Abstract

**Background:**

Rates of alcohol and/or substance use (ASU) among residents of predominantly Black and marginalized communities are similar to ASU rates in White communities. Yet ASU has worse consequences in predominantly Black and marginalized communities (e.g., higher incarceration).

**Objective:**

We randomized participants to one of 16 intervention conditions using a 2^4^ full factorial design to optimize a multilevel intervention reducing ASU among 602 formerly incarcerated men with substance-use-disorders (SUD). Candidate intervention components included (1) critical dialogue (CD; six weekly 2-hour-long group sessions vs. no CD sessions), (2) Quality of Life Wheel (QLW; six weekly 1-hour-long group sessions vs. no QLW sessions), (3) capacity building projects (CBP; six weekly 1-hour-long group sessions vs. no CBP sessions), and (4) delivery by a trained peer versus licensed facilitators. Outcome was percentage of days in which participants used alcohol, cocaine, opioid, and/or cannabis in previous 30 days.

**Results:**

Intent-to-treat analysis did not meet a priori component selection criteria due to low intervention attendance. After controlling for intervention group attendance (percentage of sessions attended), peer-delivered CD and CBP produced statistically and clinically significant main and interaction effects in ASU over 5 months. Per the multiphase optimization strategy framework, we selected peer-delivered CD and CBP for inclusion as the optimized version of the intervention with a cost of US$1,380 per 10 individuals. No adverse intervention effects occurred.

**Conclusion:**

CD and CBP were identified as the only potentially effective intervention components. Future research will examine strategies to improve attendance and test the optimized intervention against standard of care in a randomized-controlled-trial.

## Introduction

Rates of alcohol and/or substance use (ASU) in predominantly Black and marginalized communities (low-income communities with low access to quality education, employment, and housing) are similar to those in White communities in urban areas (Substance Use & Mental Health Services Administration, 2020). Nonetheless, graver consequences of ASU (e.g. higher incarceration and HIV/HCV infection rates) are found among residents of predominantly Black and marginalized communities, particularly formerly incarcerated men (Gibbons et al., 2023; Jones et al., 2023; Substance Use & Mental Health Services Administration, 2020). Such differences are partly attributed to social determinants of health (SDOH), including intersectional stigmas (e.g. prejudice against people of color, formerly incarcerated people, and people with substance use disorder [SUD]); poverty; and lack of access to quality education, safe housing, and meaningful employment (Farahmand et al., 2020). Yet, evidence-based interventions to decrease ASU have overlooked community members’ experiential knowledge and input for intervention development and have thus missed opportunities to explicitly address SDOH (Sugarman et al., 2020).

For the current project, in order to decrease ASU frequency in a population of self-identified men with histories of SUD and incarceration in Newark, NJ, United States, we collaborated with community members to optimize *Community Wise*, a multilevel manualized behavioral intervention (Windsor et al, 2014a; Windsor et al, 2018). We used the multiphase optimization strategy framework (MOST) alongside community-based participatory research (CBPR) principles and best practices (e.g. power sharing in decision-making that values experiential knowledge) to develop and optimize *Community Wise* (Collins, 2018; Israel et al., 2010). Community members were trained in the basic concepts of CBPR and MOST, notably how MOST employs experimental designs to engineer efficient and effective behavioral interventions (Collins, 2018). This enabled us to complement each other’s knowledge and skills in all stages of the research cycle. MOST allowed us to include community needs identified by community members, to employ rigorous and systematic scientific methods, and to reduce costs by selecting a design that emphasized efficiency and sustainability (Windsor et al, 2021).

### Rationale for Testing *Community Wise* for Men with Histories of Incarceration and SUD

In the United States, most formerly incarcerated individuals self-identify as men. People released from incarceration often return to predominantly Black and historically marginalized communities that have been neglected by governments and private investments resulting in insufficient social services and high rates of poverty, crime, and unemployment (Lynch et al., 2021). As a result of racism, classism, and heteronormative stereotypes of masculinity, most formerly incarcerated men face elevated socioeconomic and health-related needs, including disease prevention and health care, housing, and employment (Smiley & Fakunle, 2016; Wolff & Shi, 2010). Stigma and structural barriers lead to weak connections to the labor market which in turn, strengthen connections to illicit markets that, coupled with police profiling and racism, contribute to reincarceration (Dunlap et al., 2006). Due to the disproportionate impact of incarceration on men when compared to women and statistical requirements for sample size, we decided, in collaboration with our community partners, to focus the current project on men only, while including women in other research projects.

Despite evidence suggesting that interventions for reducing health inequities must address SDOH, most evidence-based SUD interventions focus solely on changing individual cognition and behavior (Sugarman et al., 2020). The impacts of racism, sexism, homophobia/transphobia, and classism on poor health outcomes are well documented (Farahmand et al., 2020). Research has shown that interventions emphasizing community engagement often help participants strengthen their social networks and develop critical thinking needed to address ASU (Wallerstein et al., 2020). Likewise, interventions addressing community members’ identified needs and aspirations can reduce ASU, improve health, and increase access to employment opportunities and housing while facilitating policy changes (Wallerstein & Duran, 2016).

#### Intervention Theoretical Framework and Pilot Studies

We used critical consciousness (CC) theory to inform the development of a multilevel intervention that promotes critical reflection regarding one’s own social, political, and economic conditions and critical actions (e.g. civic engagement), which has been shown to reduce the impact of SDOH and ASU (Wallerstein & Duran, 2016). Our study is the first to use CC theory to reduce ASU among formerly incarcerated men. Here we posit that CC is both a process *and* an outcome taking place at the psychological, social/relational, and community levels. We operationalized CC as participants’ (1) perceived knowledge about beliefs and norms regarding how SDOH impacts ASU-related health inequities (e.g. racism, disproportionately higher drug-related incarceration of people of color) and (2) capacity to engage in behavioral and social actions that help reduce health inequities (e.g. find treatment services, advocate for universal health services). CC development involves guided discussions focused on specific social conditions depicted in painted illustrations, a technique often used by Paulo Freire, who coined the term “critical consciousness” (Freire, 2000). We hypothesize that CC has potential to help intervention participants develop an accurate understanding of how marginalization (e.g. stigma-based social and economic exclusion) is associated with health inequities. We consider CC to be the key ingredient that can help *Community Wise* participants stay well-connected to their communities, engage in activities to address SDOH, and develop meaning in their lives through the belief that they can alter their environment in a way that helps improve personal and community health. Commitment to treatment, self-efficacy, social support, and a sense of having a purpose in life have been significant predictors of successful SUD treatments (Burleson & Kaminer, 2005).

**Figure 1.**
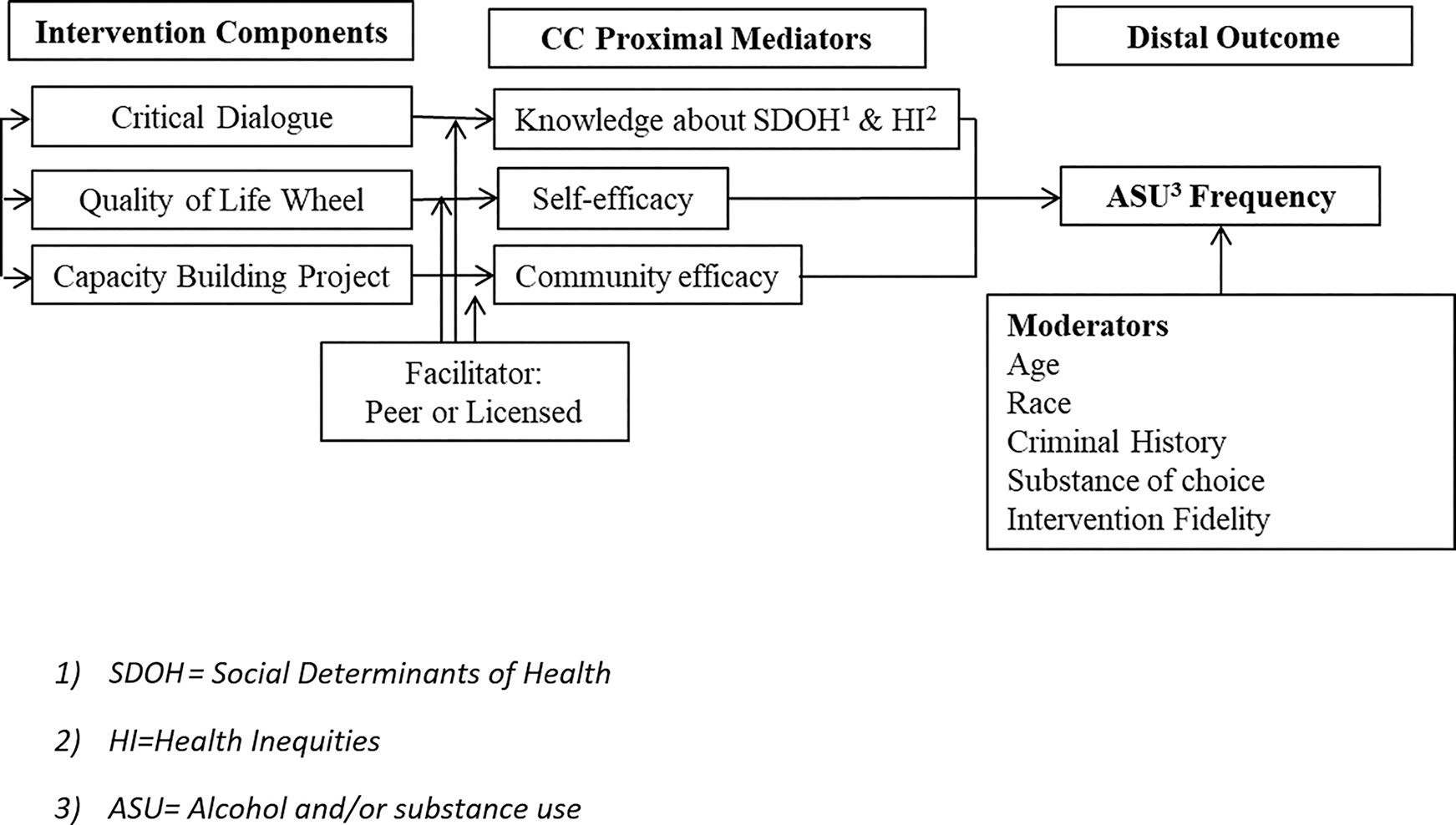
Conceptual model: *Community Wise* components to reduce alcohol and substance use among formerly incarcerated men. Note: Mediating and moderating effects were not tested in this study.

*Community Wise* pilot studies provided further support for the use of CC theory in the field of SUD treatment (Windsor et al, 2014b). Our published findings suggest pre-post intervention increases in CC and reductions in ASU outcomes. Results also suggested that *Community Wise* had high acceptability with high scores on engagement (average 7.42 out of 12 [±3.90] sessions attended) and 62% of participants successfully completing the intervention (operationalized as consistently attending sessions, making clinical improvement, and engaging in capacity-building projects [CBP]; Windsor et al, 2014b). Figure 1 displays the conceptual model that informed our selection of candidate components.

#### Community-Engaged Development of Community Wise

Adhering to CBPR principles (e.g. power-sharing, valuing community knowledge, etc.), we created the Critical Consciousness Collaborative in 2010 (3C; www.the3C.org). Through collaboration and consensus, the 3C identified SUD and related health inequities (e.g. incarceration, HIV/HCV infection) as the most important health-related issues affecting predominantly Black and marginalized communities in Newark, New Jersey (Windsor & Murugan, 2013). After selecting CC theory as an appropriate theory, we used Carroll and Nuros’ (2002) staged model of manual development to create the first version of *Community Wise* containing three components: Critical Dialogue (CD), Quality of Life Wheel (QLW), and CBP. These were delivered together in our pilot study (Windsor et al, 2014b).

*Community Wise* was designed to address SDOH at the *micro level* (e.g. cognitive and behavioral processes), the *meso level* (e.g. relationships with individuals/organizations), and the *macro level* (e.g. political and cultural processes). Blending experiential and scientific knowledge, we identified the following components to be tested for potential inclusion in the final optimized intervention: (1) CD-group discussions about SDOH and inequity, (2) QLW-development of individual goals, and (3) CBP-group projects to solve community problems. We also considered it critical to determine whether trained peer facilitators (TPF) could deliver *Community Wise* as effectively as trained licensed facilitators (TLF), as this would lower the cost of intervention delivery given lower facilitator salary base and provide employment opportunities for formerly incarcerated people to become TPFs. Thus, a fourth component (efficacy of *Community Wise* delivery by TPFs vs. TLFs) was added.

## Methods

Grounded in a robust theoretical framework and our previous findings (Windsor et al, 2014a and 2014b), the current study utilized a 2^4^ full factorial experimental design to estimate the individual and combined effects of *Community Wise’s* four intervention components in reducing ASU over a 5-month period. We hypothesized (conceptual model: Figure 1) that there would be a three-way interaction between each of the intervention components and that there would be no significant differences in reduction of ASU between participants who attended groups facilitated by TPFs and TLFs.

In following MOST principles of efficiency, we selected affordability as our constraint criterion (Collins, 2018). We agreed that affordable interventions needed to be delivered at cost allowed by Medicaid because this would increase intervention access to people living below the poverty line. Consequently, we tracked intervention delivery costs and decided a priori to include solely statistically and/or clinically significant components that could be delivered for US$225 or less per person (French et al., 2008). Table 1 shows factorial design conditions and delivery costs.

### CBPR as a paradigm for developing and testing Community Wise

*Community Wise* was developed and piloted through ongoing work of the 3C, with extensive involvement of health service providers, community stakeholders (e.g. residents and government employees), and formerly incarcerated men with a history of SUD. The collaborative has been consistently funded to meet at least once per month. Together we have published *Community Wise’s* intervention development processes, pilot findings, and evaluations of our CBPR approach (www.the3c.org). Thus, *Community Wise* benefits from sustained investment by community members and organizations nested in predominantly Black and marginalized communities (Windsor et al, 2014a). Every 3C member received training in ASU, HIV prevention, reentry issues, CC theory, intervention development, and CBPR. We value scientific and experiential knowledge, maintain open dialogue and transparency, and strive to distribute power equitably (Windsor et al, 2014a).

We grounded the current study in the scientific literature, our pilot findings, the integrated theoretical framework, and our partners’ experiential knowledge (www.the3C.org; National Institute on Drug Abuse, 2007). Collectively, we decided group delivery of *Community Wise* would be best to help individuals affected by ASU enhance their CC and engage in processes to reduce health inequities. These processes, and corresponding changes, occur at the micro, meso, and macro levels. Because of methodological limitations (e.g. lack of designs to assess an intervention’s impact on multilevel and multiple outcomes simultaneously), we optimized *Community Wise* based on a single, primary individual-level outcome, here referred to as ASU, a widely studied variable that could be evaluated over the study’s time frame of 5 months. We collected data on other network- and community-level outcomes through individual-level, self-reported measures, and qualitative data to inform future studies designed to assess the intervention’s impact on multilevel outcomes.

3C members received US$20 per hour for their work on the project. The full board met once every 2 months to receive project updates and provide feedback. 3C members organized into several task forces to support specific project functions (e.g. recruitment, measurement selection, community engagement). Moreover, the 3C maintained Governance and Sustainability committees, which oversaw 3C membership issues; funding; and decisions about bylaws, procedures, and organization of meetings.

### Multiphase Optimization Strategy

MOST is a methodological framework that emphasizes efficiency and careful resource management, while developing, optimizing and evaluating multicomponent behavioral interventions (Collins, 2018). Optimization trials, such as the current study, are conducted to identify which intervention components (and component levels), individually and in combination, are most effective before evaluation using a two-arm randomized controlled trial. MOST is well-suited for studies with significant community collaboration and partnership, as these enhance efficiency by identifying resource constraints and culturally acceptable problem-solving approaches (Windsor et el, 2021). Ours is the first community-engaged study to implement MOST to optimize a multilevel intervention in a closed group format for reducing ASU-related health inequities.

### Study Procedures

#### Setting

This project was conducted from December 2016 to December 2020 at our partner community-based organization, which has a long history of providing health and mental health services to predominantly Black and marginalized populations in Newark, NJ. All required IRB approval was obtained from the Champaign (approval #16574).

#### Recruitment and Data Collection

Recruitment strategies included indigenous field worker sampling (Platt et al., 2006), facility-based sampling, community advertisement (fliers), and street outreach. Interested individuals were encouraged to call the project’s cell phone number or visit our partner community-based organization and complete a preliminary screening with outreach workers. Potential participants meeting initial recruitment criteria were invited to individual, in-person screening interviews to establish eligibility. These interviews were conducted by trained master’s- or doctoral-level clinicians using computerized versions of the Mini Mental State Exam (Folstein et al., 2001), the Timeline Follow-Back (Sobell & Sobell, 1996), and the Global Appraisal of Individual Needs-Substance Problem Scale (Dennis et al., 2006). Participants received US$10 to complete the 40-min screening. Eligible participants completed a study orientation session and those who consented to enroll in the study completed the baseline and follow-up instruments directly *via* a tablet, with help from a research assistant. Follow-ups started 1 month after initial *Community Wise* sessions and continued for a total of 5 months. Follow-up assessments included the Timeline Follow-Back and a urine toxicology screen. Participants received cash incentives on site in paper money or *via* Cash App for completing data collection.

#### Inclusion and Exclusion Criteria

Inclusion and exclusion criteria are presented in Table 2. Eligible participants completed orientation about randomization procedures. Those deemed to understand and agree to this study’s procedures were invited to provide consent and enroll.

#### Experimental Design

We used a 2^4^ full factorial experiment to evaluate individual and interactive effects of the presence or absence of four intervention components on ASU reduction. The study included 16 experimental conditions (Table 1). Since each of the intervention components was delivered in a group setting, participants were randomized to a group (within the experimental condition). Power calculations indicated that there should be three groups (with 11 participants per group) for each experimental condition, thus requiring 48 groups in total. Due to the intervention intensity, the experiment took place over 3 years, allowing 16 groups per year. The order of delivery of experimental conditions was randomly selected a priori *via* a commercially available random number generator across the 3-year period. After collecting baseline data from 22 participants (a sufficient number to fill two intervention groups), co-investigators with no clinical contact with participants randomized participants to one of the 16 experimental conditions that were ready for delivery based on the random list. After randomization, participants were given their group meeting date and time, determined by when the majority of group members could meet.

### Intervention Components

#### Core Component

All participants received a core component including a *Community Wise* introduction, a critical reflection session, a termination session, and a graduation ceremony. The core component sets the foundation for subsequent candidate components by creating a safe space for participants to engage in dialogue (Session 1) and helps participants understand critical reflection and knowledge building (Session 2). During the termination session, participants measure their growth, review knowledge gained, and outline future action items. Graduations were public celebrations, creating a forum where the community was informed about participants’ achievements and where community members, family members, and experts celebrated the participants and developed their next steps.

#### Critical Dialogue Component

Each CD session, lasting about 2 hours, was delivered in Sessions 3, 5, 7, 9, 11, and 13. CD consisted of group conversations guided by critical questions raised by facilitators and group members and stimulated by programmatic images (Windsor, et al, 2014a). These sessions were designed to help participants analyze SDOH’s impact on their lives, behavior, and communities. *Community Wise* participants randomized to receive CD were encouraged to contemplate their beliefs and behaviors that are routinely influenced by their environment and often characterized by racist, sexist, and classist ideologies. Once aware of such influences, participants began to explore alternative interpretations of systems of oppression and their influences in their lives.

#### Quality of Life Wheel Component

The QLW approach was adapted from CC culture circles (Hope and Timmel, 2001) and was delivered in the first 60 minutes of Sessions 4, 6, 8, 10, 12, and 14. In accordance with specific, measurable, attainable, relevant, and time bound (SMART) goal setting (Bovend’Eerdt et al., 2009), participants systematically identified long-term personal goals, focusing on feasible, measurable, and specific steps. QLW was designed to increase participants’ self-efficacy and enhance future-oriented development. Participants receiving QLW reported their progress to the group and received feedback and encouragement.

#### Capacity-Building Projects Component

The CBP component drew on community organizing strategies to mobilize communities and foster positive change and was delivered in the second 60 minutes of Sessions 4, 6, 8, 10, 12, and 14. Accordingly, CBP necessitated collective action toward relationship building and improving community capacity. *Community Wise* participants randomized to receive CBP were entrusted with addressing SDOH in their community through selection, design, and implementation of a communal project of their choice. Examples of CBP included writing letters to government representatives or stakeholders to increase access to housing, fundraising to promote health, voter registration efforts, and meaningful employment for formerly incarcerated people.

#### Facilitator Type Component

TLFs or TPFs separately delivered all possible component combinations. TLFs had master’s degrees in human services and were licensed chemical and alcohol drug counselors and certified alcohol and substance abuse counselors. TPFs were unlicensed, had high school degrees, histories of SUD and incarceration, and were community members. All facilitators were Black with ties to the community. Both groups received training and ongoing clinical supervision from principal investigators.

### Measurement

#### Outcome Measures

During all five monthly follow-ups, participants self-reported alcohol consumption and completed a urine toxicology screen for opioids, heroin, cocaine, and cannabis (including synthetic). The primary outcome was ASU (i.e. the percentage of days each substance was used in the past month) as operationalized by the Global Assessment of Individual needs (Dennis et al., 2003). At each time point (baseline plus five follow-ups), ASU was calculated by dividing the reported number of days in the past month that participants used cannabis, heroin, alcohol, opioids, or cocaine by the number of days in the month (data were collected with the Timeline Follow-Back measure). We conducted correlations for self-reported ASU in the past 30 days with toxicology urine screens to assess validity of self-reported data.

#### Process Measures

Group cohesion was assessed using a 12-item Group Climate Questionnaire with a 6-point scale and a 12-item Working Alliance Inventory with a 5-point scale (Horvath & Greenberg, 1989; MacKenzie, 1983). Curative quality was evaluated with a 15-item Curative Factors Questionnaire using a 4-point scale (Yalom, 1995). Respondents were instructed to address these questions with reference to how they felt when participating in the group sessions. Scored variables for these three instruments were treated as continuous measures of group cohesion and curative quality. Participant satisfaction was assessed with a 26-item End of Treatment Questionnaire developed by the *Community Wise* optimization project team. Questions (7-point scale) focused on the helpfulness of participating in the intervention. We also asked how much the participants would use the information learned, whether they would recommend the intervention to others, and their preference for different intervention content using a sliding scale (0–100). Delivered treatment doses were assessed using attendance data collected by facilitators with binary options of Present/Absent. Percentage of attendance was calculated by dividing the number of sessions attended by the number of total sessions (this ranged from three to 15 sessions depending on experimental condition assignment) multiplied by 100.

Treatment fidelity was evaluated by a team of six graduate students not involved in treatment delivery, who reviewed 35% (*n* = 180) of randomly selected session recordings. The evaluation team assessed three areas (facilitation skills, manual adherence, and education skills), using multiple objective indicators for each domain. Intervention delivery cost was calculated for an optimized *Community Wise* group with 10 participants. This cost included the TPF’s salary (27 h; US$15/h), weekly clinical supervision (9 h; US$100/h), participant public transportation (US$3.50/person/session), and refreshments (US$20/session).

#### Data Analysis

##### Data Preparation

The data’s distributional characteristics were examined, (skewness, kurtosis, and illogical values) and descriptive summaries calculated (number and percent for categorical variables; mean [*M*], standard deviation [*SD*], median, minimum, and maximum for continuous variables) using SAS version 9.4 (SAS Institute, Inc., Cary, NC).

Covariates were included in the model based on their association with the components, ASU, group membership, and number of sessions attended (*p* < 0.05). Covariates consisted of other drug or alcohol treatment received in the past month (Yes/No), incarceration duration (months), marital status (Married vs. Other), income at baseline (continuous), number of days paid for working in the past month (continuous), race/ethnicity (Black, White, Hispanic: Yes/No), whether life was disturbed by memories or feelings of something, readiness to remain abstinent from ASU, number of days under electronic monitoring, whether participant got into trouble with their probation or parole officer, criminalized behavior score in past month, and general mental health score (Dennis et al., 2006). The criminalized behavior score was calculated by summing the number of times during the past month the participant took something from a store without paying for it; sold, distributed, or helped to make illegal drugs; drove under the influence; or had a disagreement in which they pushed, grabbed, or shoved someone. The score for general mental health was calculated by summing responses for number of days they were bothered by any psychological problems and number of days they experienced problems that kept them from meeting their responsibilities at work, school, or home.

##### Factorial Experiment Analytical Plan (Primary Analysis)

We examined the effect of each *Community Wise* intervention component (e.g. CD, QLW, CBP, and facilitator type) in reducing ASU across six time points (baseline plus five monthly follow-ups) among all enrolled individuals using SAS version 9.4 (SAS Institute, Inc., Cary, NC). A generalized linear mixed-effects (multilevel) model was used to examine associations between intervention candidate components and average percentage of ASU (repeated measures over time) as the outcome, using effect coding (received = 1; not received = −1). Facilitator capacity was coded as −1 for TPFs and 1 for TLFs. All two-, three-, and four-way interactions for candidate components were included in the model. The use of effect coding allowed for direct interpretation of the classical definition of the main and interaction effects; estimates only needed to be multiplied by a factor of 2 to determine the size of the effect (Kugler et al., 2012). Effects for random subjects nested within group and random slope (repeated measures over time) were included to account for subject variation at baseline and over time (baseline, five follow-ups). The autoregressive covariance structure was used for repeated observations. A lognormal distribution was specified for the outcome due to its skewness.

Descriptive statistics showed that the average percentage of sessions attended was 16% (SD = 26.6). Because of differences in the total number of possible sessions across experimental groups, we used the number of sessions attended as an offset to account for this difference in “exposure” as a modified intent-to-treat (ITT) analysis (Anderson et al., 2007). The extent of clustering of participants within groups over time was investigated using the intraclass correlation coefficient (ICC). This was accomplished by fitting an unconditional generalized linear mixed model to ASU as the outcome, while only adjusting for random effects for intercept, time, and random subjects nested within group. For example, the ICC for between-group effects was calculated as [variance between groups/variance between groups + variance due to autoregressive type 1 correlation within a subject over time + residual variance]. An ICC of ≥ 0.01 was considered remarkable clustering (Bliese, 1998). The ICC for the clustering of individuals was 0.03 between subjects and 0.92 between groups.

Generalizable effect size estimates (Cohen’s *d*) were calculated as the estimate × 2/pooled SD. When calculating the pooled SD, the observed sample size for each level of the component was used (e.g. sample size for those who received the CD component and sample size for those who did not receive the CD component). Criteria for considering the efficacy of a component were a Cohen’s *d* ≥ 0.25, *p*value ≤0.1, or both (Cohen, 1988). All available data, including non-completers, were analyzed in accordance with the ITT population approach, using SAS version 9.4 (SAS Institute, Inc., Cary, NC).

##### Missing Data Analyses and Sensitivity Analysis

We conducted a per protocol analysis as an additional sensitivity analysis to examine the potential impact of low attendance rates. This analysis was restricted to individuals who had at least one intervention session (*n* = 254). CD and CBP matched the criteria for retention based on the clinical significance (*p* < 0.1) and/or the effect size (*d* ≥ 0.25) in the modified ITT population, but only CD matched both criteria in the per protocol population. Full per protocol analysis results are available in Table 3.

Missing data patterns in the ASU outcome and covariate values were examined across baseline and follow-ups 2 and 5. There were 23 data pattern groups at baseline, 32 at follow-up 2, and 31 at follow-up 5. Most of the missing data groups had a very limited sample size (<30). When examining the mean values of the variables across the missing data group, no discernable trends in the values of ASU or other covariates were observed compared to the group without missing data. We also created missingness indicators for the outcome and examined their proportion per time point. Patterns of missingness were non-monotone. We examined the influence of departures from the missing at random assumption (MAR; i.e., the probability that the outcome is missing depends on the observed data) using multiple imputation with pattern-mixture models under the assumption of missing not at random (MNAR; i.e., probability that the missingness is related to the unobserved data; Iddrisu & Gumedze, 2019). Pattern-mixture models examine the distribution of an outcome as the mixture of observed and missing value patterns. Since the MNAR is not directly testable, conducting this sensitivity analysis approach enables us to compare inferential results for the missing values to results for imputed values. We considered the MAR assumption questionable if it was not robust to the MNAR assumption, that is, it generated results that differed from the results for the MNAR scenarios (van Buuren, 2007).

Data was imputed using the fully conditional specification method and the multiple imputation model included the same covariates used in the primary analysis. Following five imputations, the generalized linear mixed-effects model used for the primary analysis was fitted in each imputed dataset (i.e., each imputed dataset was analyzed separately). For inference, the model results were then combined to generate overall estimates with their associated standard error. Imputation diagnostics were examined, including the fraction of missing information and the relative efficiency for each parameter estimate. The fraction of missing information, which indicates the percentage of the total sampling variance that is attributable to missing data, ranged from 1% to 5%. Relative efficiencies were >99% for all components, indicating that the number of imputations was sufficient. Parameter estimate results are available in Table 4.

## Results

### Sample

**Figure 2.**
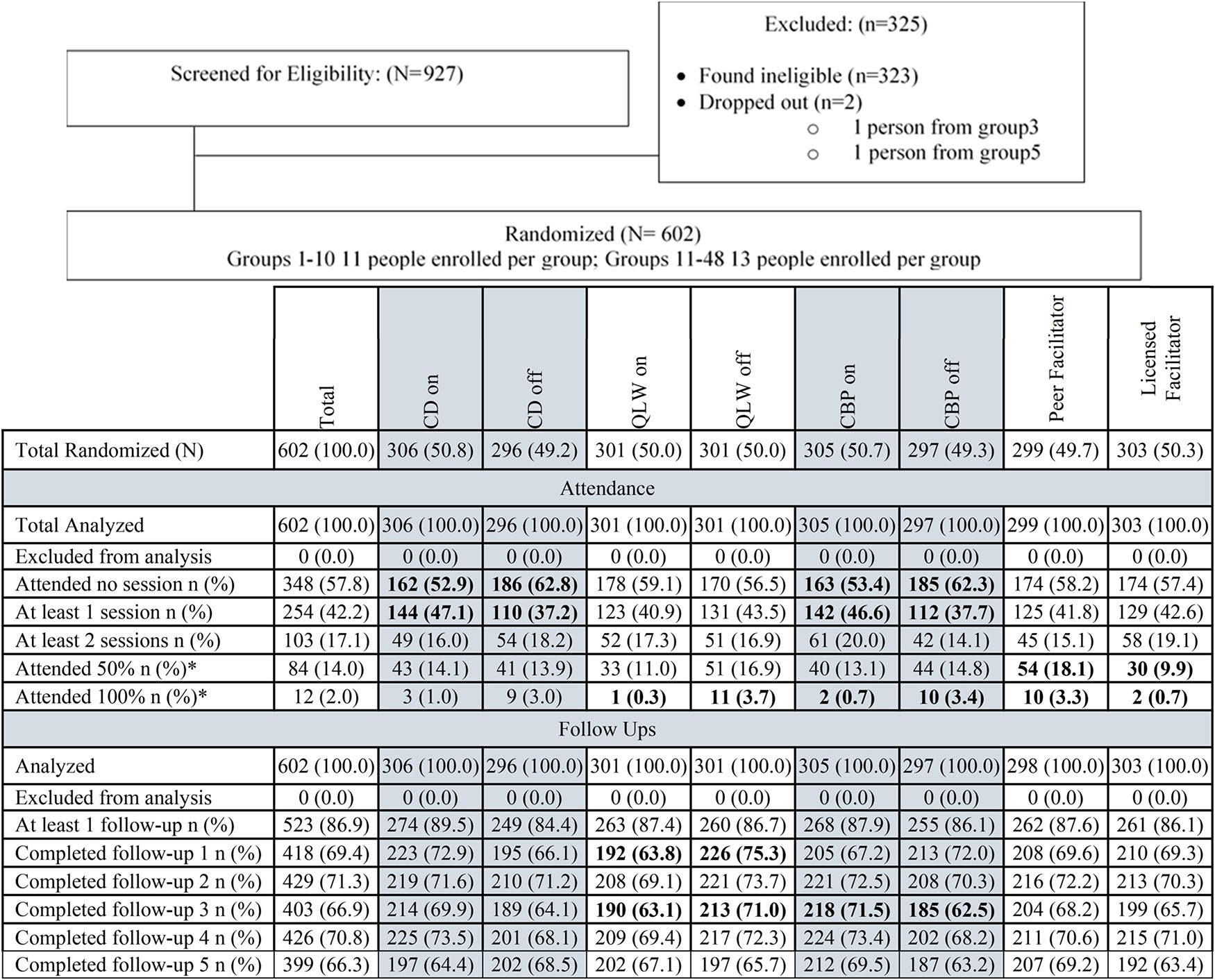
Consort Diagram for Groups. *Note.* Bolded values = Pearson chi-square tests; *p* < 0.05. *Number of sessions varied from 3 to 15.

We recruited 927 self-identified men. Out of 927 potential participants, 323 were ineligible after screening, two dropped out, and 602 (99.6%) consented to participate in the study (Figure 2). Table 5 displays participants’ demographics at baseline.

### Process Data: Attendance, Feasibility, and Intervention Fidelity

Forty-two percent of participants attended ≥1 session and 14% completed ≥50% of sessions. There were no statistically significant differences in attendance rates across experimental conditions (Figure 2). Those participating in the intervention reported high satisfaction; 85% indicated they would recommend the intervention to others and rated the intervention as being greatly helpful (*M* = 5.37, SD = 1.55; scale of 1–7). Participants rated the working alliance with facilitators highly, with a mean score of 58.74 (SD = 14.61; scale of 1–100). Treatment fidelity was also high, with facilitators using appropriate delivery skills about 81%–96% of the time. Additionally, facilitators adhered to the intervention manual 85%–95% of the time and educated participants on the intervention’s essential concepts and procedures as expected 62%–96% of the time. No adverse consequences were identified.

### ASU Outcomes

Table 6 presents descriptive summaries of raw ASU by intervention component. The intent to treat (ITT) analysis did not meet the a priori component selection criteria. This is likely a byproduct of low attendance rates. Thus, we conducted a modified ITT analysis, which included all 602 participants. Here we controlled for intervention attendance and found statistically and clinically significant effects. The modified ITT analysis results informed the selection of components for the optimized intervention. Table 7 presents results of the full model, testing effects of intervention components and of facilitator capacity and their interactions on ASU (primary outcome) over 5 months. Of the three intervention components, CD and CBP met both selection criteria (*p* < 0.1 and Cohen’s *d* ≥ 0.25). Compared to participants who did not receive CBP, those who did were observed to have a reduction in ASU averaged over 5 months of follow-up after controlling for covariates (Cohen’s *d* = −0.36, *p* = 0.026). Compared to individuals who did not receive CD, those who did were observed to have a larger reduction in mean ASU, after controlling for covariates (Cohen’s *d* = −0.65, *p* < 0.0001). The effect of CD on the outcome was observed to differ by whether individuals received CBP. The interaction effect of CD and CBP was synergistic as defined in the MOST framework with a statistically significant Cohen’s d of −0.33, *p* = 0.043 (Collins, 2018).

### Intervention Cost and Optimization Decision-Making

Following community-engaged research best practices (Wallerstein & Duran, 2016), in collaboration with our community partners we used the findings to inform the optimized version of *Community Wise* and its updated manual. As guided by the principles of MOST (Collins, 2018), we only included components that met our a priori criteria of cost, statistical significance, and main effects Cohen’s *d* that significantly reduced ASU. We considered all possible interaction effects to identify synergistic reductions in ASU and intervention costs. The CD × CBP interaction was synergistic and met the intervention cost criterion (i.e. < US$225/person), with a delivery cost of US$138 per person. Given low intervention retention as reported in Figure 2, the 3C concluded that the optimal strategy for implementing interventions was an eight-session open group format, in which CD and CBP are delivered back-to-back in each session, by TPFs, since there was no significant difference in the CD and CBP delivery by facilitator. We will use this format in future effectiveness trials and consider the intervention’s impact on community-level variables.

## Discussion

This study was designed to develop an optimized intervention for decreasing ASU among men with a history of SUD and incarceration. We conducted a 2^4^ full factorial experiment to evaluate individual and interactive effects of four intervention components on ASU reduction. We submit that the ITT analysis did not fully meet the a priori component selection criteria due to low attendance across the 15 sessions of *Community Wise*. Thus, we conducted a modified ITT analysis to control for intervention attendance. The modified ITT analysis supported inclusion of CD and CBP as viable intervention components and supported TPFs as efficient and effective facilitators. Consequently, CD combined with CBP, delivered by TPFs, is the optimized *Community Wise* format we plan to use in subsequent research.

Our research has significant implications for SUD treatment among formerly incarcerated men. While traditional SUD treatments can be moderately effective in reducing ASU among various populations (Windsor et al, 2015), *Community Wise* seems promising as an effective intervention that addresses ASU by acknowledging racism, classism, and sexism as SDOH, while simultaneously addressing community health issues. Although CD and CBP, delivered independently, met selection criteria for inclusion, combining CD and CBP produced a synergistic effect in ASU reductions. This supports our theoretical framework and existing literature showing both critical reflection and critical action as two dimensions of critical consciousness that are needed for social change (Jemal, 2018).

Future effectiveness research should test the optimized intervention against CD alone and a comparison group in reducing ASU. Community-level outcomes (e.g. community cohesion, capacity-building project successful completion) should be examined as secondary outcomes. Interestingly, QLW increased ASU, though the effect was neither statistically nor clinically significant. Main effect comparison between facilitator type showed a clinically, but not statistically significant effect with TLF showing higher reductions in ASU when compared to TPF. This significance disappeared when comparing interaction effects of CD, CBP, and facilitator type. Future qualitative research can be used to develop hypothesis about how facilitator type and demographic characteristics may impact ASU.

Attendance at community-based SUD treatment programs, defined as completing two or more sessions of an intervention, is challenging; typically, around 40% (Acevedo et al., 2015). Our study included formerly incarcerated men often struggling to meet basic housing, nutrition, and safety needs, who were hard to retain. Significant effort was made to remind participants of intervention sessions and address attendance barriers (e.g. scheduling sessions when most people were available; offering snacks), but many participants were houseless, had difficulties accessing transportation, and changed phone numbers often. For example, although bus tickets were provided, participants often could not get to the site to receive tickets.

Our research design, using randomization, posed significant barriers to attendance. For example, 22 participants needed to be enrolled before they could be randomized into experimental groups and before setting dates and times for group meetings. Randomization locked participants into specific groups, so scheduling conflicts could not be resolved by allowing participants to switch to a different group that met at a more convenient time. Additionally, it often took more than 30 days, from baseline to completion, to set up group start dates. This likely contributed to decreased participation due to loss of contact or interest. In comparison to the current study and similar studies, our *Community Wise* pilot study had high completion rates likely because it did not include randomization, wait time, or inflexible group meeting dates (Windsor et al, 2014b). In our future work, we plan to minimize research design barriers by using open group formats and by providing wrap-around services (e.g. transportation and ongoing communication).

Limitations of the current study included low attendance rates that likely reduced the potential effects of *Community Wise* and may have influenced the nonsignificant effects of the ITT analysis and study results. Also, we only included self-identified men in the study. It is critical to conduct comparable studies with people who identify within the continuum of gender identities and expressions. Future research is needed to replicate these findings and expand their generalizability to other regions, predominantly Black and marginalized communities, and outcomes. Nevertheless, our research supports the application of CC theory to the field of SUD, and we have future plans to examine whether CC mediates the impact of our optimized *Community Wise* intervention on ASU.

## Conclusion

Our current research combined the MOST and CBPR frameworks to identify intervention components that maximize ASU reduction among men with histories of incarceration and SUD. Using a modified ITT analysis to control for low intervention engagement, we successfully identified intervention components that reduced ASU frequency; CD and CBP yielded promising effects. The optimized *Community Wise* intervention delivered by TPFs is ready to be tested for effectiveness in community settings. We plan additional research to increase intervention engagement, test the generalizability of our findings in other settings and populations, and develop effective implementation strategies.

## Figures and Tables

**Table 1.** Experimental conditions and cost of delivery per group.

Facilitator	Experimental Condition	Core	Critical Dialogue (CD)	Quality of Life Wheel (QLW)	Capacity Building Project (CBP)	# sessions	Delivery cost*

Licensed facilitator	1: CD alone	**Present**	**Present**	Absent	Absent	9	$2,420.00
	2: QLW alone	**Present**	Absent	**Present**	Absent	6	$2,005.00
	3: CBP alone	**Present**	Absent	Absent	**Present**	6	$2,005.00
	4: CD + QLW	**Present**	**Present**	**Present**	Absent	12	$2,810.00
	5: CD + CBP	**Present**	**Present**	Absent	**Present**	12	$2,810.00
	6: QLW + CBP	**Present**	Absent	**Present**	**Present**	9	$2,420.00
	7: CD + QLW + CBP	**Present**	**Present**	**Present**	**Present**	15	$3,200.00
	8: Core only	**Present**	Absent	Absent	Absent	3	$1,640.00
Peer facilitator	9: CD alone	**Present**	**Present**	Absent	Absent	9	$1,925.00
	10: QLW alone	**Present**	Absent	**Present**	Absent	6	$1,700.00
	11: CBP alone	**Present**	Absent	Absent	**Present**	6	$1,700.00
	12: CD + QLW	**Present**	**Present**	**Present**	Absent	12	$2,150.00
	13: CD + CBP	**Present**	**Present**	Absent	**Present**	12	$2,150.00
	14: QLW + CBP	**Present**	Absent	**Present**	**Present**	9	$1,925.00
	15: CD + QLW + CBP	**Present**	**Present**	**Present**	**Present**	15	$2,375.00
	16: Core only	**Present**	Absent	Absent	Absent	3	$1,475.00

* Per 10 individuals.

**Table 2.** Inclusion and exclusion criteria.

Inclusion	Exclusion

Age ≥18 years	Gross cognitive impairment
Self-identified as male	
Agreed to being recorded during CW^a^ group sessions	Severe unstable mental illness (e.g. untreated psychotic disorder, suicidality)
Living in Newark, NJ	
Released from incarceration ≤4 years	
ability to read, write, and speak English	
Willing to provide informed consent	

a. CW, *Community Wise*.

**Table 3.** Per Protocol analysis: generalized linear mixed model examining effects of components on percentage of alcohol and substance use (%) over 5 months (*n* = 254).

Effect	Effect	*t*	*P*	Cohen’s *d*

CD alone	−1.85	−3.28	**0.001**	**−0.83**
QLW alone	−0.26	−0.44	0.660	−0.11
CBP alone	−0.56	−0.95	0.343	−0.24
CD + QLW	0.65	1.09	0.274	**0.27**
CD + CBP	0.16	0.27	0.790	0.07
QLW + CBP	0.52	0.91	0.364	0.23
CD + QLW + CBP	0.56	0.96	0.337	0.24
Licensed Facilitator (vs. Peer) alone	0.72	1.24	0.215	**0.31**
Licensed Facilitator + CD	−0.10	−0.17	0.866	−0.04
Licensed Facilitator + QLW	−0.57	−0.96	0.336	−0.24
Licensed Facilitator + CBP	0.10	0.18	0.859	0.04
Licensed Facilitator + CD + QLW	−0.08	−0.14	0.886	−0.04
Licensed Facilitator + CD + CBP	0.75	1.29	0.197	**0.33**
Licensed Facilitator + QLW + CBP	0.29	0.5	0.616	0.13
Licensed Facilitator + CD + QLW + CBP	0.57	0.98	0.325	**0.25**

Regression effects assume a log-normal distribution of the outcome and model the effect of the component relative to individuals who did not receive the component. Given the use of effect coding, the regression effects are calculated as 2 X parameter estimate from the regression model. Effects that meet the criteria for selection (*p* < 0.1 or absolute value of Cohen’s *d* ≥ 0.25) are bolded. Cohen’s d was considered a generalizable effect size estimate and calculated as the ratio of the effect to the pooled standard deviation. Components abbreviations: CD = Critical Dialogue, QLW = Quality of Life Wheel, CBP = Capacity Building Project.

**Table 4.** Generalized linear mixed model examining effects of components and facilitator type on percentage of alcohol and substance use (%) over 5 months following multiple imputation of missing data (*n* = 602).

Effect	Effect	*t*	*P*	Cohen’s *d*

*CD alone*	−1.15	−4.76	**0.00**	**−0.03**
*QLW alone*	0.13	0.51	0.61	0.16
*CBP alone*	−0.52	−2.08	**0.04**	**−0.39**
*CD + QLW*	0.34	1.39	0.16	0.07
*CD + CBP*	−0.60	−2.46	**0.01**	−0.14
*QLW + CBP*	0.57	2.38	**0.02**	**0.39**
*CD + QLW + CBP*	−0.10	−0.42	0.67	−0.07
*Licensed Facilitator (vs. Peer) alone*	0.47	1.95	**0.05**	0.03
*Licensed Facilitator + CD*	−0.05	−0.20	0.84	−0.22
*Licensed Facilitator + QLW*	−0.23	−0.96	0.34	−0.03
*Licensed Facilitator + CBP*	−0.04	−0.16	0.87	−0.03
*Licensed Facilitator + CD + QLW*	−0.33	−1.35	0.18	−0.22
*Licensed Facilitator + CD + CBP*	0.21	0.88	0.38	0.14
*Licensed Facilitator + QLW + CBP*	−0.05	−0.21	0.83	−0.03
*Licensed Facilitator + CD + QLW + CBP*	0.37	1.53	0.13	**0.25**

Multiple imputation with pattern-mixture model was applied over 5 imputations. The imputed data were analyzed as for the primary analysis using generalized linear mixed models for each imputation. Results were then integrated to examine model effects. Regression effects assume a log-normal distribution of the outcome and model the effect of the component relative to individuals who did not receive the component. Given the use of effect coding, the regression effects are calculated as 2 X parameter estimate from the regression model. Effects that meet the criteria for selection (*p* < 0.1 or absolute value of Cohen’s *d* ≥ 0.25) are bolded. Cohen’s d was considered a generalizable effect size estimate and calculated as the ratio of the effect to the pooled standard deviation. Components abbreviations: CD = Critical Dialogue, QLW = Quality of Life Wheel, CBP = Capacity Building Project.

**Table 5.** Participants’ characteristics at baseline (N = 602).

Characteristic	N(%) / Mean (±SD)

Heterosexual	542 (90.0%)
Age	45.11 (11.30)
Race	
Black/African American	485 (80.5%)
White	34 (5.6%)
Ethnicity	
Hispanic	46 (7.6%)
Not Hispanic	556 (92.4%)
Household yearly income	$5,865.02 ($1,8273.9)
Religion	
Christian	319 (53.0%)
Muslim/islam	152 (25.4%)
Jewish	4 (0.7%)
None	104 (17.3%)
Never married	402 (66.8%)
Unemployed	468 (78.9%)
Under supervision (parole, drug court, halfway house)	498 (82.7%)
Number of months since release from last incarceration	14.34 (15.3)
Substance use (mean # of days in past 30 days)	
Alcohol use	9.98 (11.6)
Cannabis use	5.63 (10.6)
Cocaine use	4.45 (9.0)
Heroin use	9.39 (12.8)
Opiate use	1.13 (5.0)
Prevalence of current substance use	
Alcohol	402 (66.8%)
Cannabis	211 (35.0%)
Cocaine	202 (33.6%)
Heroin	256 (42.5%)
Opiate	49 (8.1%)
Received other SUD treatment	263 (43.7%)
Received welfare support	189 (31.4%)
Homeless living in the street	81 (13.5%)
Lived in structured living situation	8 (1.3%)
Lived in homeless shelter	224 (37.2%)
Mental health	
Traumatic Stress Scale (TSS)	4.93 (3.99)
Somatic Symptom index (SSI)	1.51 (1.5)
Depressive Symptom index (DSS)	3.27 (3.18)
Length of incarceration (in month)	9.6 (24.5)
Criminal Justice System index (CJSI)	7.08 (17.15)
Readiness for abstinence (out of 100)	71.44 (30.84)

**Table 6.** Description of raw average percentage of alcohol and substance use (ASu) over time (*N* = 602).

	Average percentage of ASU in past 30 days (median [min, max])											
	B	F1	F2	F3	F4	F5	B	F1	F2	F3	F4	F5

Total	20 (0,100)	13 (0,70)	17 (0,58)	16 (0,80)	18 (0,73)	17 (0,100)	--	--	--	--	--	--
By intervention component			Received						Did not Receive			
Critical Dialogue (CD)	20 (0,100)	13 (0,53)	16 (0,53)	16 (0,51)	15 (0,52)	15 (0,80)	20 (0,100)	14 (0,70)	18 (0,58)	16 (0,80)	20 (0,73)	20 (0,100)
Quality of Life Wheel (QLW)	20 (0,80)	13 (0,70)	17 (0,53)	16 (0,51)	15 (0,54)	20 (0,100)	20 (0,100)	14 (0,57)	17 (0,58)	17 (0,80)	20 (0,73)	17 (0,80)
Capacity Building Project (CBP)	20 (0,100)	13 (0,57)	17 (0,58)	15 (0,57)	16 (0,60)	19 (0,100)	20 (0,100)	14 (0,70)	17 (0,51)	17 (0,80)	20 (0.73)	17 (0,80)
By facilitator type			Peer Facilitator						Licensed Facilitator			
	20 (0,100)	13 (0,57)	15 (0,58)	15 (0,47)	17 (0,60)	17 (0,100)	19 (0,83)	14 (0,70)	20 (0,52)	18 (0,80)	20 (0,73)	18 (0,100)

B = baseline; F1-F5 = follow-up (post-baseline) months 1 to 5. No differences were observed by intervention component or facilitator.

**Table 7.** Modified intent to treat analysis: generalized linear mixed model examining effects of components on percentage of ASU (%) over 5 months (*N* = 602).

Effect	Effect	*t*	*p*	Cohen’s *d*

CD alone	−1.21	−3.96	**<0.0001**	**−0.65**
QLW alone	0.25	0.80	0.424	0.13
CBP alone	−0.68	−2.23	**0.026**	**−0.36**
CD + QLW	0.28	0.91	0.364	0.15
CD + CBP	−0.62	−2.02	**0.043**	**−0.33**
QLW + CBP	0.56	1.83	**0.067**	**−0.29**
CD + QLW + CBP	−0.09	−0.28	0.776	−0.05
Licensed Facilitator (vs. Peer) alone	0.49	1.62	0.105	**0.26**
Licensed Facilitator + CD	−0.19	−0.62	0.538	−0.10
Licensed Facilitator + QLW	−0.10	−0.34	0.735	−0.06
Licensed Facilitator + CBP	−0.13	−0.43	0.668	−0.07
Licensed Facilitator + CD + QLW	−0.24	−0.81	0.419	−0.13
Licensed Facilitator + CD + CBP	0.01	0.04	0.965	0.01
Licensed Facilitator + QLW + CBP	0.18	0.60	0.552	0.09
Licensed Facilitator + CD + QLW + CBP	0.25	0.81	0.416	0.13

*Note*. Regression effects assume a log-normal distribution of the outcome and model the effect of the component relative to individuals who did not receive the component. Given the use of effect coding, the regression effects are calculated as 2 X parameter estimate from the regression model. Effects that meet the criteria for selection (*p* < 0.1 or absolute value of Cohen’s *d* ≥ 0.25) are bolded. Cohen’s *d* was considered a generalizable effect size estimate and calculated as the ratio of the effect to the pooled standard deviation. Components abbreviations: CD = critical dialogue, QLW = Quality of Life Wheel, CBP = capacity-building project.

## Data Availability

Due to confidentiality and privacy concerns, the data will not be deposited for public access. Those interested in obtaining the data can contact the corresponding author to request the data and sign a data sharing agreement.
